# Biomaker evaluation for major adverse cardiovascular event development in patients undergoing cardiac Surgery

**DOI:** 10.1515/almed-2020-0031

**Published:** 2020-07-27

**Authors:** Claudia E. Imperiali, Juan C. Lopez-Delgado, Macarena Dastis-Arias, Lourdes Sanchez-Navarro

**Affiliations:** Clinical Laboratory, Hospital Universitari de Bellvitge. L’Hospitalet de Llobregat, Barcelona, Spain; Biochemistry and Molecular Biology Department, Universitat Autonoma de Barcelona, Barcelona, Spain; Critical Care Unit, Hospital Universitari de Bellvitge. L’Hospitalet de Llobregat, Barcelona, Spain

**Keywords:** biomarkers, cardiac surgery, outcome

## Abstract

**Objectives:**

The postoperative period of cardiac surgery (CS) is associated with the development of major adverse cardiovascular events (MACEs). However, the evaluation of MACE after CS by means of biomarkers is poorly developed. We aimed to evaluate postoperative biomarkers that could be associated with MACE.

**Methods:**

Two Hundred and ten patients who underwent CS were enrolled during the study period. The diagnosis of MACE was defined as the presence of at least one of the following complications: acute myocardial infarction, heart failure, stroke presented during intensive care unit (ICU) stay, and 30-day mortality after CS. High-sensitive troponin T (hs-TnT), C-reactive protein, procalcitonin, interleukin-6, and immature platelet fraction (IPF) were measured on ICU admission and after 24 h. The difference between both measurements (Δ) was calculated to assess their association with MACE. Early infected patients (n=13) after CS were excluded from final analysis.

**Results:**

The most frequent surgery was single-valve surgery (n=83; 38%), followed by coronary artery bypass graft (n=72; 34%). Postoperative MACE was diagnosed in 31 (14.8%) patients. Biomarker dynamics showed elevated values at 24 h compared with those at ICU admission in patients with MACE versus no-MACE. Multivariate analysis showed that ΔIPF (OR: 1.47; 95% CI: 1.110–1.960; p=0.008) and Δhs-TnT (OR: 1.001; 95% CI: 1.0002–1.001; p=0.008) were independently associated with MACE.

**Conclusions:**

These findings suggest that postoperative ΔIPF and Δhs-TnT may be useful biomarkers for the identification of patients at risk of MACE development.

## Introduction

Cardiac surgery (CS) is a major surgery associated with substantial morbidity and mortality [1]. Despite improvements in anesthesiology, surgical procedures and postoperative care, cardiovascular complications still represent an important cause of postoperative morbidity and mortality [2]. Thus, the occurrence of major adverse cardiovascular events (MACE) as a complication after major surgery is not infrequent [3]. The definition of MACE involves several of those cardiovascular complications: acute myocardial infarction, stroke, heart failure, and also mortality itself after a cardiovascular procedure [4].

Several risk factors, such as age, gender, and comorbidities, have been used to assess the risk of MACE in patients who undergo surgery. However, MACE is not just a consequence of these risk factors or preexisting comorbidities, many intraoperative variables may influence the development of postoperative complications, and therefore MACE development [5]. The inflammatory response that occurs as a consequence of an intense surgical procedure and prolonged cardiopulmonary bypass might play a key role in the development of adverse cardiovascular events after CS, emphasizing the close relationship between inflammation and outcomes [6], [7]. Some biomarkers may have a potential role in MACE prediction: cardiac biomarkers such as high-sensitive troponin I or T (hs-TnI/T) have been demonstrated to be stronger predictors of adverse cardiovascular outcomes [8], whereas some inflammatory biomarkers, such as C-reactive protein (CRP), procalcitonin (PCT), and interleukin-6 (IL-6), have a controversial association with MACE although it seems that there may be a strong rationale [9], [10], [11], [12], [13].

Most recently, immature platelet fraction (IPF), also called reticulated platelets, has been gained interest as a potential predictor of MACE after surgery [5], [14], [15]. They are metabolically and enzymatically more active than mature platelets and have a greater prothrombotic activity, which is closely related with decompensated inflammatory status [16]. In addition, IPF has recently shown an association between inflammatory response and outcomes after CS [17]. In consequence, it seems likely that IPF may be associated with MACE after CS. The usefulness of the IPF is based on the fact that its measurement has been currently integrated into the routine hematology analyzer, making the IPF value available in a generalized way.

In agreement with previous data we hypothesized that the cardiac and inflammatory biomarkers could be associated with the development of cardiovascular outcome and mortality after CS. Therefore, our study was aimed to evaluate the association between postoperative biomarkers, such as hs-TnT, CRP, PCT, IL-6, and IPF with MACE development in the setting of CS.

## Materials and methods

### Patients

This is a prospective and observational single-center study performed in a third-level university affiliated hospital, from March 2016 to March 2017. The study was approved by the local Ethics Committee of the hospital (Reference No. PR090/16). Written informed consent was obtained from each patient prior to enrollment. This study was conducted in accordance with the Declaration of Helsinki and standards of good clinical practice.

Patients were eligible for the study if they were ≥ 18 years old and underwent CS with an intensive care unit (ICU) stay of at least 48 h. CS included the following surgical procedures: coronary artery bypass graft (CABG), valve surgery, aortic surgery, and cardiac tumor resection. Exclusion criteria were as follows: preexisting immunosuppression, hematological and oncological diseases in the last 5 years, and heart transplantation.

The operations were performed throughout the study period by the same group of cardiac surgeons using median sternotomy, standard cardiopulmonary bypass with moderate hypothermia (> 34 °C), and antegrade cardioplegia. A mean aortic pressure of > 65 mmHg was maintained during CS. For revascularization, the internal thoracic artery (or bilateral if possible) and saphenous vein grafts were used. Bypass graft flow was assessed for each graft by Doppler transit time flowmetry. All patients were transferred to the cardiac ICU postoperatively. All patients received prophylactic antibiotics after surgery based on local protocols. In all patients, decisions regarding postoperative ICU management were made by the attending physician according to local protocols.

Data were extracted from the electronic medical records of each patient and collected in a database for analysis purposes. Preoperative data (i.e., demographic data, comorbidities, and treatment before surgery), operative data, and postoperative variables were routinely collected.

### Methods

The main study outcome was the presence of MACE during the follow-up period. MACE was recorded if any of the described complications were present from ICU admission immediately after surgery to ICU discharge and mortality at 30 days after CS. The diagnosis of MACE was defined as, at least, the presence of one of any of the following complications: acute myocardial infraction, heart failure, stroke, and any all-cause mortality. Acute myocardial infarction, heart failure, and stroke were defined according current definitions and clinical practice [18], [19], [20], [21], [22], [23], [24]. Briefly, acute myocardial infarction was defined as the presence of abnormal repolarization on electrocardiogram (i.e., Q waves, ST-segment elevation or depression, new left bundle branch block), imaging evidence of new regional wall motion abnormality and elevation of cardiac biomarkers [18], [20]. Stroke was defined as the presence of an acute episode of a focal or global neurological deficit [21], [22]. Heart failure was defined as the appearance of a recent deterioration in heart function or the worsening in patients with previous heart failure, and the need of inotropes or vasopressors to keep hemodynamic stability during postoperative period [19], [23]. An echocardiography, or invasive monitoring in the most severe cases (i.e., pulmonary artery catheter), was performed at the bedside in order to confirm the diagnosis of postoperative heart failure [24].

#### Blood collection and measurement of biomarkers

Blood samples for the study were taken by central venous catheter at ICU admission postoperatively and then at 24 h postoperatively. Plasmas were collected in 4 mL tubes containing Lithium Heparin (Vaccuette®, Greiner Bio One®, Kremsmünster, Austria). After collection, samples were centrifuged at 2000 rpm for 10 min and immediately processed and/or stored at −20 °C until measurement. The hs-TnT and CRP were measured immediately on Cobas 6000 analyzer (Roche Diagnostics International, Rotkreuz, Switzerland) using the electrochemical luminescent method and immunoturbidimetric method, respectively. IL-6 and PCT were measured from stored plasma on Cobas 6000 analyzer using the electrochemical luminescent method.

For IPF measurement, samples were collected at ICU admission and 24 h after CS in 4 mL tubes containing tripotassium ethylenediaminetetraacetic acid (Vaccuette®, Greiner Bio One®, Kremsmünster, Austria) and stored at room temperature before measurement. All samples were analyzed within 2–4 h after collection using Sysmex® XN (Sysmex®, Kobe, Japan) according to the manufacturer’s recommendations.

Additionally, preoperative and postoperative data related to renal and hepatic function assessment (creatinine, total bilirubin, and alanine aminotransferase) were measured on Cobas 6000 using spectrophotometry method. Hemoglobin, leucocytes, and platelets concentrations were measured on Sysmex XN analyzer. Hemoglobin was measured by means of sodium lauril sulfate photometric method and leukocytes and platelets by fluorescence flow cytometry method with semiconductor diode laser.

The Acute Physiology and Chronic Health Evaluation II (APACHE II) score was calculated for each patient during the first 24 h of ICU stay.

#### Statistical analysis

Continuous variables are presented as means and standard deviations or medians and interquartile ranges as appropriate. The significance of the differences of the mean between groups was assessed using a Student’s t-test if the distributions of the data were normal or the Mann–Whitney U test for variables with skewed distributions. For categorical variables, we used the Pearson χ^2^ test or Fisher’s exact test, where appropriate.

A logistic regression model was performed to examine the association between biomarkers (hs-TnT, CRP, IL-6, PCT, and IPF) and MACE development (outcome variable).

The established risk factors of age, sex, intraoperative transfusion, and aortic cross-clamping time (ACC) were considered to be potential covariates for the model. Only the variables with statistical significance in the univariate model were included in the final model. A probability of ≤ 0.05 was considered statistically significant. Statistical analyses were performed using Stata/MP, version 14 (StataCorp LP).

## Results

During the study period, 210 adult patients (n=120) were included. Demographics and preoperative data of all patients and grouped according to MACE development are shown in Table 1.

**Table 1:** Baseline characteristics of all patients and subgroups.

Characteristics	Patients (n=210)	No MACE (n=179)	MACE (n=31)	p-Value
Male sex, n (%)	137 (65)	120 (67)	17 (57.8)	0.188
Age, years (min–max)	70 (28–86)	70 (28–86)	71 (72–85)	0.566
BMI, kg/m^2^, mean ± SD	30 ± 4.8	29 ± 4.9	27.6 ± 3.5	0.120
Laboratory parameters				
Hemoglobin, g/L, mean ± SD	132 ± 16	132 ± 19	131 ± 18	0.847
Leukocytes, × 10^9^, mean ± SD	8.0 ± 2.3	8.1 ± 2.3	7.4 ± 1.9	0.173
Platelets count, ×10^9^/L, mean ± SD	218 ± 63	222 ± 63	188 ± 52	0.009
Creatinine, µmol/L, mean ± SD	94 ± 31	94 ± 32	95 ± 25	0.413
Total bilirubin, µmol/L, mean ± SD	10 ± 6	9.7 ± 5.2	12.7 ± 10.9	0.332
ALT, U/L, median (IQR)	18.6 (13.8–25.8)	18.6 (13.8–25.2)	20.4 (15–28.8)	0.447
Comorbidity and therapy				
Diabetes mellitus, n (%)	85 (40.5)	73 (40.8)	12 (38.7)	0.828
Hypertension, n (%)	165 (78.6)	141 (78.8)	24 (77.4)	0.866
Dyslipidemia, n (%)	138 (65.7)	119 (66.5)	19 (61.3)	0.574
COPD, n (%)	19 (9.5)	15 (8.4)	4 (12.9)	0.418
NYHA class III and IV, n (%)	73 (34.8)	58 (32.4)	15 (48.4)	0.198
Previous MI, n (%)	61 (29)	49 (27.4)	12 (38.7)	0.199
Previous stroke, n (%)	22 (10.5)	16 (8.9)	6 (19.4)	0.080
Aspirin, n (%)	104 (49.5)	90 (50.3)	14 (45.2)	0.599
Beta-blocker, n (%)	112 (53.3)	95 (53.1)	17 (54.8)	0.856
Statin, n (%)	134 (63.8)	114 (64)	20 (64.5)	0.960

Data are presented as number (%) or mean ± SD or median (IQR) according with data distribution. Years are presented as a mean (minimum-maximum). BMI, body mass index; ALT, alanine aminotransferase; COPD, chronic obstructive pulmonary disease; NYHA, New York Heart Association; MI, myocardial infraction.

The median ICU stay was 3 (2–5) days and the mean APACHE II was 12.9 ± 4.6. At least one outcome event of the MACE definition was diagnosed in 31 (14.8%) patients (Table 2). More than one outcome was occurred in five patients. Two patients showed heart failure (HF) and death. One patient developed HF and myocardial infraction (MI) and another patient presented HF and stroke. Finally, the last patient presented HF, MI, and death.

**Table 2:** Outcome events presented after CS.

Event	n (%)
MI, n (%)	6 (2.9)
Stroke, n (%)	2 (0.95)
HF, n (%)	22 (10.5)
Death 1-month, n (%)	7 (3.3)
At least one MACE, n (%)	31 (14.8)

Data are presented as number (%). MI, myocardial infraction; HF, heart failure; CS, cardiac surgery; MACE, major adverse cardiovascular event.

The most frequent surgery was single-valve surgery (n=83; 38%), followed by CABG (n=72; 34%), mixed procedures (CABG plus valve: n=22; 10.5%), double valve (n=17; 8%), aortic surgery (n=11; 5.3%), and miscellaneous, which mainly included myxoma extraction (n=5; 2.4%). The remaining surgical parameters are shown in Table 3.

**Table 3:** Surgical data of total patients and in patients with and without MACE.

Surgical parameters	Total	No MACE	MACE	p-Value
	(n=210)	(n=179)	(n=31)	
Surgical procedure, n (%)				
CABG	72 (34.2)	64 (35.8)	8 (25.8)	0.075
Valve surgery	83 (39.5)	74 (41.3)	9 (29)	
Double valve	17 (8.1)	13 (7.3)	4 (12.9)	
Mixed procedures	22 (10.5)	15 (8.4)	7 (22.6)	
Aortic surgery	11 (5.2)	8 (4.5)	3 (9.7)	
Miscellaneous	5 (2.4)	5 (2.8)	0	
Elective surgery, n (%)	155 (73.8)	135 (75.4)	20 (64.5)	0.184
Operating time, min, mean ± SD	277 ± 88	272.5 ± 84	305.9 ± 106	0.085
CPB time, min, mean ± SD	103 ± 38	100.6 ± 36	114.3 ± 46	0.115
ACC time, min, mean ± SD	61.8 ± 37	58.2 ± 36	82.7 ± 36	0.004
Intraoperative blood cells transfusion, n (%)	38 (18.1)	26 (14.5)	12 (38.7)	0.001
Number of blood cells, n (%)				0.008
1	20 (9.5)	14 (7.8)	6 (19.4)	
2	15 (7.1)	9 (5)	6 (19.4)	
3	2 (1)	2 (1.1)	0	
4	1 (0.5)	1 (0.06)	0	
Intraoperative platelets transfusion, n (%)	24 (11.4)	15 (8.4)	9 (29)	0.001
Number of platelet pools, n (%)				<0.001
1	19 (9.1)	14 (7.8)	5 (16.1)	
2	5 (2.4)	1 (0.6)	4 (12.9)	

Data are presented as number (%), mean ± SD. CABG, coronary artery bypass grafting; CPB, cardiopulmonary bypass; ACC, aortic-cross clamping; MACE, major adverse cardiovascular event.

### Biomarkers

Biomarkers (hs-TnT, CRP, IL-6, PCT, and IPF) were measured immediately at ICU admission and 24 h after CS. IL-6 and PCT at 24 h were measured only in 137 (65.2%) of the total patients due to a lack of plasma separation or not refrigerated immediately after extraction, which can affect IL-6 stability [25]. Measurement of the remaining biomarkers was routinely performed at both times. The delta of each biomarker was calculated for each patient. It was defined as the difference between day 1 after CS and the day of ICU admission. For IPF (%), the difference is expressed in percentage points.

Biomarker dynamics showed elevated values in patients with MACE compared to those without MACE. However, not all biomarkers were significantly higher in patients with MACE than those without MACE (Table 4). Only the delta of PCT, hs-TnT, and IPF was statistically significantly higher in MACE compared to no-MACE. The relative percentage change of each biomarker was 661% for PCT, −3.8% for hs-TnT, and 18% for IPF in no-MACE and 2.975%, 48%, and 37.5%, respectively, in MACE. Since the presence of infection could cause higher biomarker concentrations, especially of CRP, IL-6, PCT, and IPF, the data were analyzed excluding patients who developed infection during the first 3 days after CS (Table 4). In total, 13 patients presented with infection. The most frequent infection was pneumonia (n=10; 79.9%), followed by mediastinitis (n=1; 7.7%), surgical wound infection (n=1, 7.7%), and urinary tract infection (n=1; 7.7%). Furthermore, the remaining postoperative laboratory results related to renal, hepatic, and hemogram parameters, are shown in Table 5.

**Table 4:** Biomarkers concentrations in patients with and without MACE.

	Time	All patients						Excluding early infection				
		n	Total patients	no MACE^a^	MACE^b^	p-Value		n	Total patients	no MACE^c^	MACE^d^	p-Value
PCT, median (IQR), µg/L	0	210	0.057 (0.037–0.09)	0.056 (0.034–0.09)	0.08 (0.046–0.16)	**0.025**		197	0.056 (0.035–0.09)	0.055 (0.034–0.090)	0.08 (0.045–0.101)	0.064
	24	137	0.501 (0.233–1.68)	0.425 (0.19–1.09)	2.46 (0.67–5.34)	**<0.001**		128	0.43 (0.216–1.635)	0.415 (0.186–1.08)	2.175 (0.69–4.64)	**0.002**
Delta of PCT, µg/L		137	0.414 (0.149–1.547)	0.373 (0.136–1.01)	2.054 (0.624–5.25)	**<0.001**		128	0.387 (0.141–1.409)	0.362 (0.131–0.955)	1.937 (0.645–4.60)	**0.001**
IL-6, median (IQR), ng/L	0	210	183.2 (112.9–315)	177.5 (108.7–282.9)	228.8 (153.5–621)	**0.010**		197	181.3 (111.8–291.8)	174.4 (106.2–282.75)	218.7 (159–404.5)	**0.032**
	24	137	160.2 (104.3–241.5)	156.1 (102.2–228.1)	241.5 (107.2–508.6)	0.057		128	156.1 (103–234.2)	156.1 (102.2–227.3)	161.1 (104.3–421.4)	0.502
Delta of IL-6, ng/L		137	23.2 (−158.6–59.7)	−5.53 (−142.4 to 59.7)	−87.4 (−382.6 to 172.8)	0.462		128	−15.3 (−142.6 to 59.7)	−5.5 (−130.4 to 59.7)	−64.4 (−215.4 to 51.5)	0.614
CRP, median (IQR), mg/L	0	210	2.3 (1–4.5)	2.3 (1–4.6)	2.5 (0.8–4.1)	0.998		197	2.3 (1–4.5)	2.3 (1–4.5)	2.2 (0.8–4.1)	0.802
	24	210	148.4 (103.4–201)	146.1 (103.4–196.5)	167.6 (97.5–227.6)	0.348		197	149.1 (96.4–199.6)	147.4 (97.5–196.1)	164.4 (88.5–225.9)	0.542
Delta of CRP, mg/L		210	145.3 (93.8–199)	142.4 (94.7–192)	163 (90.3–223)	0.5		197	145.6 (92.3–200.8)	142.4 (92.9–192.1)	172.9 (80.4–222.5)	0.057
hs-TnT, median (IQR), ng/L	0	210	415 (250.5–795)	390 (241–708)	626 (387–1631)	**0.001**		197	400 (249–730)	387 (240–706)	576 (369–1344)	**0.013**
	24	210	409 (265–719)	375 (249–579)	926 (490–1580)	**<0.001**		197	388 (255–674)	366 (246–374)	828 (423–1244)	**<0.001**
Delta of hs-TnT, ng/L		210	−14.8 (−246 to 133)	−31.9 (−248 to 72)	139 (−45.5 to 583)	**0.003**		197	−18.8 (−246.3 to 115)	−35.4 (−249 to 72)	120 (−6 to 461)	**0.006**
IPF, median (IQR), %	0	210	4 (2.6–5.4)	3.9 (2.6–5.4)	4 (2.7–5.4)	0.547		197	3.9 (2.6–5.3)	3.9 (2.6–5.4)	4 (3.2–5.3)	0.696
	24	210	4.7 (3.2–6.6)	4.6 (3.1–6.5)	5.5 (4.1–9.1)	**0.025**		197	4.6 (3.1–6.5)	4.6 (3–6.5)	5.3 (4.1–7.1)	0.151
Delta of IPF PP		210	0.7 (0–1.5)	0.6 (0–1.4)	1 (0.1–2.5)	**0.048**		197	0.5 (0–1.3)	0.5 (−0.1–1.3)	1 (0.1–1.9)	**0.014**

Data are presented as median (IQR). PCT, procalcitonin; IL-6, interleukin-6; CRP, C-reactive protein; hs-TnT, high sensitive T troponin; IPF, immature platelet fraction; PP, percentage points. ^a^n=179, ^b^n=31, ^c^n=172, ^d^n=25. At 24 h after CS for PCT, IL-6 and their delta values: ^a^n=118, ^b^n=19, ^c^n=114, ^d^n=14.

**Table 5:** Postoperative laboratory results.

Postoperative (ICU admission)	Patients (n=210)	No MACE (n=179)	MACE (n=31)	p-Value
Hemoglobin, g/L, mean ± SD	106 ± 15	107 ± 15	101 ± 15	0.129
Leukocytes, ×10^9^/L, mean ± SD	13.1 ± 5.5	13.0 ± 5.6	13.0 ± 4.6	0.590
Platelets count, ×10^9^/L, mean ± SD	164 ± 57	168 ± 58	143 ± 46	0.022
Creatinine, µmol/L, mean ± SD	79 ± 32	79 ± 34	81 ± 23	0.250
Total bilirubin, µmol/L, mean ± SD	15 ± 9	15 ± 8	19 ± 11	0.038
ALT, U/L, median, IQR	17 (13–25)	16 (13–24)	20 (14–31)	0.066
Postoperative (24 after CS)				
Hemoglobin, g/L, mean ± SD	101 ± 14	101 ± 13	98 ± 16	0.405
Leukocytes, ×10^9^/L, mean ± SD	14.4 ± 4.3	14.1 ± 4.1	16.3 ± 5.1	0.019
Platelets count, ×10^9^/L, mean ± SD	166 ± 54	169 ± 56	148 ± 48	0.073
Creatinine, µmol/L, mean ± SD	90 ± 50	87 ± 47	112 ± 61	0.004
Total bilirubin, µmol/L, mean ± SD	13 ± 9	12 ± 9	17 ± 14	0.013
ALT, U/L, median, IQR	19 (15–29)	18(14–25)	34 (22–102)	<0.001

Data are presented as number (%) or mean ± SD or median (IQR) according with data distribution. ALT, alanine aminotransferase; MACE, major adverse cardiovascular event.

### Logistic regression

The univariate analysis exposed differences between patients with and without MACE with respect to ACC time, transfusion of hemoderivatives, delta of troponin and delta of IPF. In contrast, age and sex were not different (Table 6). After accounting for ACC time and transfusion of hemoderivatives, the association of delta of IPF and delta of hs-TnT with the occurrence of MACE remained significant. On the one hand, the delta of IPF demonstrated the strongest independent association with a highest OR of 1.47 (CI: 1.11–1.96). On the other hand, the delta of hs-TnT also shows an independent association with an OR of 1.001 (CI: 1.0002–1.001). This result represents the risk of developing MACE was found to be increased by 10% for a rise of the delta of hs-TnT of 100 ng/L (Table 6).

**Table 6:** Logistic regression model of variables associated with MACE occurrence.

	Univariate		Multivariate	
Variables	OR (95% CI)	p-Value	OR (95% CI)	p-Value
Sex	1.67 (0.77–3.63)	0.191	–	–
Age	1.02 (0.98–1.06)	0.380	–	–
ACC time	1.02 (1.01–1.03)	0.001	1.02 (1.01–1.03)	0.003
Transfusion of hemoderivates	2.77 (1.45–5.32)	0.002	1.97 (0.90–4.28)	0.089
ΔIPF	1.55 (1.17–2.07)	0.002	1.47 (1.11–1.96)	0.008
ΔPCT	1.10 (0.97–1.24)	0.124	–	–
Δhs-TnT	1.001 (1.0002–1.002)	0.010	1.001 (1.0002–1.001)	0.008

OR, odds ratio; CI, confidence interval; ACC time, Aortic cross-clamping; ΔIPF, delta of immature platelet fraction; ΔPCT, delta of procalcitonin; Δhs-TnT, delta of high-sensitive troponin T.

## Discussion

Despite surgical advances and improvements in the postoperative management of CS patients, cardiovascular complications after CS are an important issue that can worsen patients’ outcomes. In this prospective study, with more than 200 patients who underwent CS, we found that the delta of IPF, and the delta of hs-TnT to a lesser extent, are both associated with postoperative MACE development, independently of the presence of established risk factors.

Our findings may demonstrate the potential usefulness of IPF and hs-TnT to detect increased risk of MACE development after CS. This result was in concordance with previous studies. Multiples studies have shown the association of hs-TnI/T with MACE [26], [27], [28]. To date, only one published study found a relationship between IPF and MACE, but this study was performed in non-CS patients [5]. To the best of our knowledge, there is no study showing this association in CS patients until now.

CS may exacerbate or enhance the inflammatory response, which has important clinical implications leading to postoperative heart failure. Many biochemical and molecular mechanisms are involved in the pathogenesis of the inflammatory response: nitric oxid alteration generation, increased levels of proinflammatory cytokines, and endothelium dysfunction, among others. As a consequence, the inflammatory response after CS may increase the risk of a postoperative cardiac event as well a variety of organs dysfunction [29].

Since an inflammatory response could be involved in the pathogenesis of cardiovascular outcomes after CS, we hypothesized that inflammatory biomarkers such as PCT, CRP, IL-6, and IPF could be associated with MACE development.

As expected, on the one hand, we found that some laboratory parameters related with renal, hepatic, and also leukocytes, are higher during postoperative period in patients who developed MACE compared to those who did not. MACE is closely linked with inflammatory response. Likewise, this inflammatory response contributes to the pathogenesis of organ failure [29].

On the other hand, in our study, we found that at least one of the criteria of MACE was present in 31 patients. No differences were found between types of surgical procedures and MACE development. Regarding biomarkers, all studied biomarkers demonstrated higher values in the MACE group, with statistically significant differences in delta values for PCT, hs-TnT, and IPF. No differences between groups were found for the deltas of IL-6 and CRP. It is well-known that PCT, IL-6, CRP, and also IPF are strongly related with infections; hence, we also examined the results after excluding patients who developed an infection during the first 3 days after surgery.

The behavior of the deltas of different biomarkers were the same even after excluding patients with early postoperative infections. Regarding IL-6, a recent study performed in adult CS found a significant association with IL-6 levels and mortality following CS [30]. In contrast with our study, they studied 1-year mortality.

Despite differences in CRP, the delta not being statistically significant, its behavior showed the same trend (positive) in both groups. CRP results were lower at ICU admission compared to CRP levels 24 h after surgery, which may be related to the kinetics of this acute-phase protein. Early postoperative CRP levels start to increase within 2 h and achieve their peak after 48 h [31].

A major surgical procedure always triggers a significant inflammatory response, and CRP increases after CS as a response to inflammation induced by surgery trauma in an unspecific way, which may ultimately explain why we did not find differences between groups. CRP does not represent a useful biomarker in this setting because of its elevation after an uncomplicated course.

Some studies have reported higher PCT levels in patients with complications after CS. Liu et al. [32] found a PCT peak within 24 h postoperatively, noting that PCT concentrations and PCT clearance were significantly higher in the non-survivor group compared with survivor group after CS. Clementi et al. [13] found that PCT concentrations measured at 48 h after CS were significantly higher in renal and respiratory outcomes and in septic patients, and in agreement with our study, they did not find differences in cardiovascular outcomes either between patients who died during hospitalization, at 30 days or at 6 months.

Troponin T or I are cardiac biomarkers that reflect the pathophysiological mechanism of MACE from a direct myocardial injury point of view. In a large cohort of coronary artery disease patients, elevated hs-TnI levels were associated with an increased risk of MACE [8]. Another study conducted by Mauermann et al. [26] examined the association of troponin changes from first and second postoperative days in CS patients with all-cause mortality and cardiac morbidity, finding that an increase of more than 10% in troponin levels was significant associated with the endpoint. All these findings are in accordance with our study, which were found even after cross-clamping time multivariate analysis adjustment.

Research on IPF has recently focused on the potential association with clinical outcomes in patients with cardiovascular diseases and major surgery. While IPF production is known to be promoted by low platelet concentration in blood, its role in inflammatory conditions is still less understood and the mechanism for increased IPF levels after surgery have not been well explored so far. Growing evidence supports the fact that the pathophysiological mechanism through which IPF increases after inflammatory stimuli is related with thrombopoietin regulation in response to acute-phase mediators. IL-6 increases thrombopoietin transcription from the liver and consequently thrombopoietin rises causing a reactive platelet production [33].

Previous studies demonstrated the association of IPF with inflammatory response complications but only one of these was performed in CS patients [17]. IPF levels may be useful as a biomarker for the early identification of patients at risk of inflammatory response development after CS. The role of IPF in infection has been widely studied. Previous research revealed its utility especially in septic patients. Park et al. [34] found that IPF has high sensitivity and accuracy in the discrimination of septic from nonseptic patients. Moreover, Koyama et al. [35] reported that immature platelet count is associated with major mortality in septic patients. However, publications regarding IPF association with complications after surgery are scarce. Our data are in agreement with a study performed by Anetsberger et al. [5]. These authors found that high postoperative IPF levels are associated with an increased risk of major adverse cardiovascular as well as thromboembolic events in patients who underwent non-CS.

### Limitations of the study

The first limitation of our study is related to the sample size. The sample size limited the number of variables that could be examined to determine the independent association of biomarkers with MACE. Second, follow-up data from PCT and IL-6 at 24 h after surgery are lacking in an important number of patients due to the stability of IL-6 over the course of time. Third, only two biomarkers measurement were performed postoperatively (at ICU admission and at 24 h after CS), no further measurements were performed. However, we think that the delta between these two times could provide reliable and more early information than other intervals as, for example, ICU admission and 48 h after CS.

Finally, preoperative IPF measurement was not performed. However, a previous study performed in non-cardiac surgeries, which investigated the association with IPF and MACE, did not find significant differences in preoperative IPF results between groups [5].

## Conclusions

In summary, increased levels of the deltas of IPF and hs-TnT between 0 h and at 24 h after CS were associated with worse clinical outcomes related with adverse cardiovascular events during ICU stay and mortality at 30 days in our population. Our research shows that the deltas of IPF and hs-TnT remain associated with MACE development even after intraoperative factor adjustment, such as cross-clumping time and transfusion of hemoderivatives. Our data suggest that IPF and troponin postoperative measurement would be helpful for MACE assessment in patients undergoing CS. These data are of potential interest as IPF is an easily and inexpensively obtained routine laboratory test that is readily incorporated into clinical practice.

## References

[1] Hill A, Nesterova E, Lomivorotov V, Efremov S, Goetzenich A, Benstoem C (2018). Current evidence about nutrition support in cardiac surgery patients-what do we know?. Nutrients.

[2] Devereaux PJ, Goldman L, Cook DJ, Gilbert K, Leslie K, Guyatt GH (2005). Perioperative cardiac events in patients undergoing noncardiac surgery: a review of the magnitude of the problem, the pathophysiology of the events and methods to estimate and communicate risk. Can Med Assoc J.

[3] Sabate S, Mases A, Guilera N, Canet J, Castillo J, Orrego C (2011). Incidence and predictors of major perioperative adverse cardiac and cerebrovascular events in non-cardiac surgery. Br J Anaesth.

[4] Myles PS (2014). Meaningful outcome measures in cardiac surgery. J Extra Corpor Technol.

[5] Anetsberger A, Blobner M, Haller B, Schmid S, Umgelter K, Hager T (2017). Immature platelets as a novel biomarker for adverse cardiovascular events in patients after non-cardiac surgery. Thromb Haemost.

[6] Corral-Velez V, Lopez-Delgado JC, Betancur-Zambrano NL, Lopez-Sune N, Rojas-Lora M, Torrado H (2015). The inflammatory response in cardiac surgery: an overview of the pathophysiology and clinical implications. Inflamm Allergy Drug Targets.

[7] Shaefi S, Mittel A, Klick J, Evans A, Ivascu NS, Gutsche J (2018). Vasoplegia after cardiovascular procedures-pathophysiology and targeted therapy. J Cardiothorac Vasc Anesth.

[8] Wong YK, Cheung CYY, Tang CS, Hai JSH, Lee CH, Lau KK (2019). High-sensitivity troponin I and B-type natriuretic peptide biomarkers for prediction of cardiovascular events in patients with coronary artery disease with and without diabetes mellitus. Cardiovasc Diabetol.

[9] Kelly D, Khan SQ, Dhillon O, Quinn P, Struck J, Squire IB (2010). Procalcitonin as a prognostic marker in patients with acute myocardial infarction. Biomarkers.

[10] Brocca A, Virzi GM, de Cal M, Giavarina D, Carta M, Ronco C (2017). Elevated levels of procalcitonin and interleukin-6 are linked with postoperative complications in cardiac surgery. Scand J Surg.

[11] Twine CP (2017). The relationship between CRP and MACE: controversial and confounded. Eur J Vasc Endovasc Surg.

[12] Zhang LJ, Li N, Li Y, Zeng XT, Liu MY (2018). Cardiac biomarkers predicting MACE in patients undergoing noncardiac surgery: a meta-analysis. Front Physiol.

[13] Clementi A, Virzi GM, Mucino-Bermejo MJ, Nalesso F, Giavarina D, Carta M (2019). Presepsin and procalcitonin levels as markers of adverse postoperative complications and mortality in cardiac surgery patients. Blood Purif.

[14] Ibrahim H, Schutt RC, Hannawi B, DeLao T, Barker CM, Kleiman NS (2014). Association of immature platelets with adverse cardiovascular outcomes. J Am Coll Cardiol.

[15] Lev EI (2016). Immature platelets: clinical relevance and research perspectives. Circulation.

[16] Liu QH, Song MY, Yang BX, Xia RX (2017). Clinical significance of measuring reticulated platelets in infectious diseases. Medicine (Baltimore).

[17] Imperiali CE, Lopez-Delgado JC, Dastis-Arias M, Sanchez-Navarro L (2019). Evaluation of the delta of immature platelet fraction as a predictive biomarker of inflammatory response after cardiac surgery. J Clin Pathol.

[18] Thygesen K, Alpert JS, White HD (2007). Joint ESC/ACC/AHA/WHF task force for the redefinition of myocardial infarction. Universal definition of myocardial infraction. Eur Heart JJ Am Coll Cardiol.

[19] Lomivorotov VV, Efremov SM, Kirov MY, Fominskiy EV, Karaskov AM (2017). Low-cardiac-output syndrome after cardiac surgery. J Cardiothorac Vasc Anesth.

[20] Bianchi CF (2018). Working toward an evidence-based cutoff recommendation for myocardial infarction detection after cardiac surgery. J Thorac Cardiovasc Surg.

[21] Gaudino M, Rahouma M, Di Mauro M, Yanagawa B, Abouarab A, Demetres M (2019). Early versus delayed stroke after cardiac surgery: a systematic review and meta-analysis. J Am Heart Assoc.

[22] Cropsey C, Kennedy J, Han J, Pandharipande P (2015). Cognitive dysfunction, delirium, and stroke in cardiac surgery patients. Semin CardioThorac Vasc Anesth.

[23] Perez Vela JL, Martin Benitez JC, Carrasco Gonzalez M, de la Cal Lopez MA, Hinojosa Perez R, Sagredo Meneses V (2012). Clinical practice guide for the management of low cardiac output syndrome in the postoperative period of heart surgery. Med Intensiva.

[24] Amabili P, Benbouchta S, Roediger L, Senard M, Hubert MB, Donneau AF (2018). Low cardiac output syndrome after adult cardiac surgery: predictive value of peak systolic global longitudinal strain. Anesth Analg.

[25] Gong Y, Liang S, Zeng L, Ni Y, Zhou S, Yuan X (2019). Effects of blood sample handling procedures on measurable interleukin 6 in plasma and serum. J Clin Lab Anal.

[26] Mauermann E, Bolliger D, Fassl J, Grapow M, Seeberger EE, Seeberger MD (2017). Association of troponin trends and cardiac morbidity and mortality after on-pump cardiac surgery. Ann Thorac Surg.

[27] Mauermann E, Bolliger D, Fassl J, Grapow M, Seeberger EE, Seeberger MD (2017). Postoperative high-sensitivity troponin and its association with 30-day and 12-month, all-cause mortality in patients undergoing on-pump cardiac surgery. Anesth Analg.

[28] Humble CAS, Huang S, Jammer I, Bjork J, Chew MS (2019). Prognostic performance of preoperative cardiac troponin and perioperative changes in cardiac troponin for the prediction of major adverse cardiac events and mortality in noncardiac surgery: a systematic review and meta-analysis. PLoS One.

[29] Laffey JG, Boylan JF, Cheng DC (2002). The systemic inflammatory response to cardiac surgery: implications for the anesthesiologist. Anesthesiology.

[30] Everett AD, Alam SS, Owens SL, Parker DM, Goodrich C, Likosky DS (2019). The association between cytokines and 365-day readmission or mortality in adult cardiac surgery. J Extra Corpor Technol.

[31] Santonocito C, De Loecker I, Donadello K, Moussa MD, Markowicz S, Gullo A (2014). C-reactive protein kinetics after major surgery. Anesth Analg.

[32] Liu H, Luo Z, Liu L, Yang XM, Zhuang YM, Zhang Y (2017). Early kinetics of procalcitonin in predicting surgical outcomes in type A aortic dissection patients. Chin Med J.

[33] Kaushansky K (2005). The molecular mechanisms that control thrombopoiesis. J Clin Invest.

[34] Park SH, Ha SO, Cho YU, Park CJ, Jang S, Hong SB (2016). Immature platelet fraction in septic patients: clinical relevance of immature platelet fraction is limited to the sensitive and accurate discrimination of septic patients from non-septic patients, not to the discrimination of sepsis severity. Ann Lab Med.

[35] Koyama K, Katayama S, Muronoi T, Tonai K, Goto Y, Koinuma T (2018). Time course of immature platelet count and its relation to thrombocytopenia and mortality in patients with sepsis. PLoS One.

